# Trends in Hispanic and non-Hispanic white cesarean delivery rates on the US-Mexico border, 2000-2015

**DOI:** 10.1371/journal.pone.0203550

**Published:** 2018-09-05

**Authors:** Jill A. McDonald, Anup Amatya, Charlotte C. Gard, Jesus Sigala

**Affiliations:** 1 Department of Public Health Sciences, New Mexico State University, Las Cruces, New Mexico, United States of America; 2 Southwest Institute for Health Disparities Research, New Mexico State University, Las Cruces, New Mexico, United States of America; 3 Economics, Applied Statistics and International Business Department, New Mexico State University, Las Cruces, New Mexico, United States of America; Case Western Reserve University, UNITED STATES

## Abstract

**Background:**

Cesarean delivery occurs in one in three US births and poses risks for mothers and infants. Hispanic cesarean rates were higher than non-Hispanic white rates in the US in 2016. In 2009, cesarean rates among Hispanics on the US-Mexico border exceeded rates among US Hispanics. Since 2009, rates have declined nationwide, but border Hispanic rates have not been studied.

**Objective:**

To compare cesarean delivery rates and trends in Hispanics and non-Hispanic whites in border and nonborder counties of the four US border states before and after 2009.

**Study Design:**

We used data from birth certificates to calculate percentages of cesarean deliveries among all births and births to low-risk nulliparous women during 2000–2015, and among births to low-risk women with and without a previous cesarean during 2009–2015. We calculated 95% confidence intervals around rates and used regular and piecewise linear regression to estimate trends for four ethnic-geographic subpopulations defined by combinations of Hispanic ethnicity and border-nonborder status.

**Results:**

Of the four subpopulations, border Hispanic rates were highest every year for all cesarean outcomes. In 2015 they were 38.3% overall, 31.4% among low-risk nulliparous women, and 21.1% and 94.6% among low-risk women without and with a previous cesarean, respectively. Nonborder Hispanic rates in 2015 were lowest for all outcomes but repeat cesarean. Rates for all four subpopulations rose steadily during 2000–2009. Unlike rates for non-Hispanic whites, border and nonborder Hispanic rates did not decline post-2009. Most of the border Hispanic excess can be attributed to higher cesarean rates in Texas.

**Discussion:**

Border Hispanic cesarean rates remain higher than those among other Hispanics and non-Hispanic whites in border states and show no signs of declining. This continuing disparity warrants further analysis using individual as well as hospital, environmental and other contextual factors to help target prevention measures.

## Introduction

In 1996, one in five American babies was born by cesarean delivery (CD). By 2009, the proportion was nearly one in three [1]. This increase generated concern because CD, while critically important in some circumstances, increases the risks of complications for both mothers and infants [2]. Mothers with CDs are at increased risk of infection, hemorrhage, other serious medical and psychological complications, and hospital readmission. Newborns born via CD are more likely to have respiratory complications and less likely to be successfully breastfed [2].

The American College of Obstetricians and Gynecologists (ACOG) issued opinions in 2007 and 2009 intended in part to reduce unnecessary CD [3,4]. The California Maternal Quality Care Collaborative issued a comprehensive white paper on eliminating non-medically indicated deliveries in preterm babies in 2012 [2]. Changes in hospital policies were advocated [5], and education campaigns were launched [6]. ACOG and the Society for Maternal-Fetal Medicine issued guidelines regarding prevention of CD, noting that there was still significant concern that cesarean section was being overused [7,8]. Despite these efforts, 31.9% of all babies in the US were born by CD in 2016 [9]. This represents a small decline from the peak rate of 32.9% in 2009 [1].

CD rates in the US vary by demographic factors, including maternal race and ethnicity. Rates among US Hispanic mothers have historically been lower than non-Hispanic white (NHW) rates [10]. In 2009, however, US NHW rates began to decline while Hispanic rates continued to climb, reaching 32.2% in 2013 and surpassing NHW rates that year. Since 2013, Hispanic rates have fallen slightly, plateauing at 31.7% in 2015 and 2016, while NHW rates have continued to decline to 30.9% in 2016 [11].

**Fig 1:**
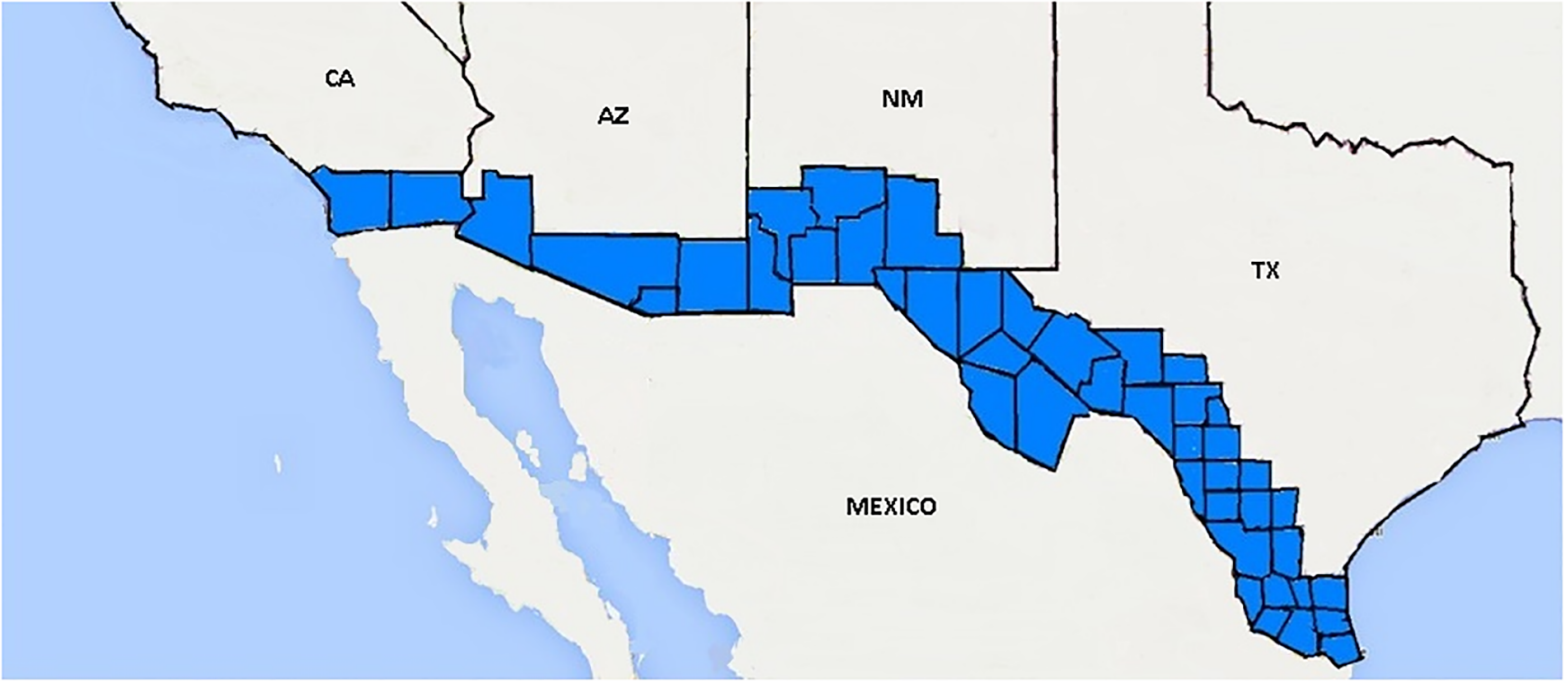
US-Mexico border region, highlighting the 44 US border counties in Arizona, California, New Mexico, and Texas.

Of particular concern are Hispanic women living in the US-Mexico border region, the set of 44 counties in the four states along the Mexican border (Fig 1). Border Hispanic women have had CD rates well above those of other racial-ethnic groups in the US. In 2009, the CD rate was 37.9% among Hispanics living in the border region as compared to 31.6% among Hispanics [12], 35.4% among non-Hispanic blacks, and 32.8% among non-Hispanic whites in the US [1]. Even among border Hispanic mothers with no prior live births, the CD rate in 2009 was 35.9% as compared to only 29.1% in the nonborder region of the border states [12]. In Texas, the disparity between border and nonborder Hispanic CD rates was even larger [13].

The aim of this study was to perform a descriptive analysis of trends in CD in the border region that builds on earlier work by adding data from 2010–2015, a critical period during which the upward trend in CDs nationally began to decline [1]. In addition to overall CD rates, our analysis examined CD outcomes among three categories of low-risk women: nulliparous, those without a previous CD (primary CD), and those with a previous CD (repeat CD). We performed this analysis for births among both Hispanic and NHW mothers in border and nonborder counties of the four border states. We determined that, unlike NHW rates in border and nonborder counties, border and nonborder Hispanic CD rates overall did not decline after 2009. Among border Hispanics, this was largely because of static rates of repeat CDs.

## Materials and methods

### Study subpopulations

The source of study data was the National Vital Statistics System (NVSS) US Standard Certificate of Live Birth files, 2000 to 2015. Records in this analysis included the 16,500,652 deidentified records of births to mothers who resided and gave birth in the US-Mexico border states of Arizona, California, New Mexico, and Texas. We selected births to mothers who reported Hispanic or NHW race-ethnicity according to prescribed NVSS methods [14]. We further classified births as border or nonborder, according to whether the mother resided in one of the 44 US-Mexico border counties [15]. We separated Hispanic and NHW births into border and nonborder populations to form four study subpopulations: border Hispanic (BH), border non-Hispanic white (BNHW), nonborder Hispanic (NBH) and nonborder non-Hispanic white (NBNHW).

### Definition of outcomes

The occurrence of a CD was based on the “Final route and method of delivery” checkbox field on the birth certificate. The CD rate, defined as the number of CDs per 100 live births, was determined for all births and for three low-risk pregnancy classifications. Low-risk was defined as term (gestational age ≥37 weeks), singleton gestation, and vertex presentation. To determine gestational age, we used the obstetric estimate or, if missing, gestational age derived from the date of the last normal menses. The 2003 revision of the birth certificate and the 1989 revision that it replaced, which were both used by border states during the study period, define vertex presentation differently [16,17]. We classified any non-breech presentation in the 1989 revision as vertex and any “cephalic” or “other” presentation in the 2003 revision as vertex, consistent with the approach of the National Center for Health Statistics [17].

**Fig 2:**
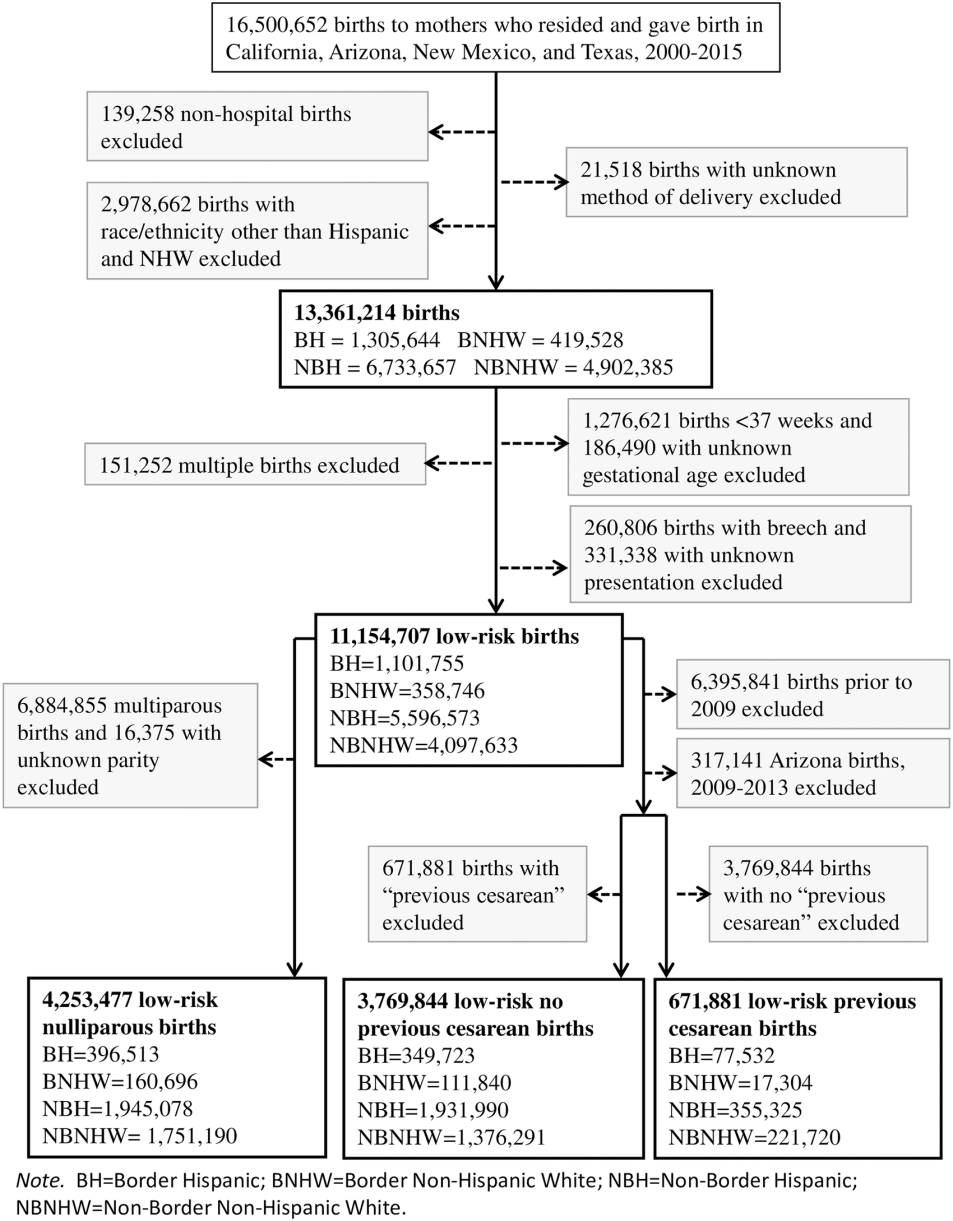
Flowchart of study population and derivation of denominators for cesarean delivery outcomes, US-Mexico border states, 2000–2015. CD, cesarean delivery; NHW, non-Hispanic white; BH, border Hispanic; BNHW, border non-Hispanic white; NBH, nonborder Hispanic; NBNHW, nonborder non-Hispanic white.

Of the 16,500,652 births in the border states during 2000–2015, we excluded 139,258 (0.8%) non-hospital births, 21,518 (0.1%) births with unknown method of delivery, and 2,978,662 (18.1%) births that were not to Hispanic or non-Hispanic white mothers, leaving a study population of 13,361,214 births (Fig 2). We calculated annual overall CD rates for the BH, BNHW, NBH and NBNHW subpopulations for 2000–2015.

To identify low-risk women who delivered a term, singleton infant with a vertex presentation, we first excluded three groups of births: 1,463,111 (10.9%) that were preterm or unknown gestational age, 151,252 (1.1%) that were multiple or unknown plurality births, and 592,144 (4.4%) that were nonvertex or unknown presentations, leaving 11,154,707 low-risk births overall. We then defined three categories of low-risk women: low-risk nulliparous women, i.e. those in their first pregnancy; low-risk women with no previous CD; and low-risk women with a previous CD. Denominators for low-risk nulliparous CD rates excluded 6,901,230 births to mothers with a previous live birth or unknown parity, leaving a total of 4,253,477 low-risk nulliparous births during 2000–2015.

Denominators for the other two low-risk CD outcomes, primary CD and repeat CD, were derived from information about previous CD on the birth certificate. Such information was first recorded on the 2003 revision of the birth certificate. However, the 2003 revision was not adopted by a majority of border states (California, New Mexico, and Texas) until 2008 and by Arizona until 2014. Therefore, to calculate primary and repeat rates, we first excluded births for all four border states prior to 2009 and for Arizona from 2009–2013. Then, to calculate denominators for primary CD rates, we excluded the 671,881 births to mothers with a previous cesarean irrespective of parity; and, conversely, to calculate denominators for repeat CD rates, we excluded the 3,769,844 births to mothers with no previous cesarean. We calculated annual overall CD rates and low-risk nulliparous rates for 2000–2015 and annual low-risk primary and low-risk repeat rates for 2009–2015.

### Maternal characteristics

We characterized CDs in each ethnic-geographic subpopulation with additional maternal variables from the birth certificate files: age, education, birth country, Hispanic origin, marital status, month of first prenatal care (PNC) visit, payment source, state of residence, parity, and risk status. We recoded the month PNC began into trimesters, including women with no prenatal care in the third-trimester group. Mothers whose birth country was a US state or territory were classified as born in the US.

Unknown values for maternal characteristics such as parity and country of birth generally occurred in <1% of records. Larger proportions of missing data occurred for some variables, e.g., payment source and prenatal care trimester, because the variables either were not included on the 1989 revision of the birth certificate or were included on the 2003 revision but were not reported by the National Center for Health Statistics (NCHS).

### Analysis

We compared the characteristics of BH mothers with CD to those of BNHW mothers, NBH mothers, and NBNHW mothers using chi-square tests. Statistical analyses of trends before and after 2009 were performed for CD rates overall and for low-risk births to nulliparous women using piecewise linear regression models, which incorporated two different linear trends in the rates of CD and allowed for a change in directionality in 2009. Analyses of trend for low-risk primary and low-risk repeat CD were performed using regular linear regression models, based on data from 2009 forward. We also conducted analyses of trend by age-group (<20, 20–34 and 35+) for each outcome and by state for CD overall. Parameters of both the piecewise and regular regression models were estimated using the ordinary least squares method. Trends in rates of CD and their 95% CIs were estimated from the fitted models and analyzed to identify differences among the study subpopulations. Student’s t-tests were used to determine the statistical significance of the estimated trends and their differences. Annual rates presented in text and tables are observed rates rather than rates estimated from the models. All analyses were performed using SAS 9.4 (Cary, NC). This study was reviewed and approved by the Institutional Review Board at the authors’ institution. The data were de-identified, so participant consent was not possible.

## Results

For the years 2000–2015, the study included 4,081,469 CDs. Of these, BH, BNHW, NBH, and NBNHW accounted for 11.2%, 3.1%, 47.7% and 38.0%, respectively. In the same years, 2,961,527 (72.6%) CDs were classified as low-risk deliveries. Among low-risk CDs, 1,094,947 (37.0%) occurred among nulliparous women. For the years 2009–2015, 1,295,027 CDs were classified as low-risk. Of these, 673,355 (52.0%) were primary CDs, and 621,672 (48.0%) were repeat CDs.

All comparisons of maternal characteristics between BH mothers having CDs during 2000–2015 and the other three study subpopulations were statistically significant (p<0.0001) unless noted in table footnotes (Table 1). In general, Hispanic mothers having CDs in border and nonborder counties were younger, less educated, more likely to be unmarried, more likely to be on Medicaid, and less likely to be nulliparous than NHW mothers in border and nonborder counties. BH mothers having CDs, compared with NBH mothers, were more likely to receive late or no prenatal care, less likely to report any kind of insurance, and much more likely to live in Texas (65.0% BH versus 33.1% NBH in Texas). BH mothers having CDs were more likely to have early term deliveries (33.4%) and to be classified as low-risk (79.8%) than any other study subpopulation. Finally, BH mothers were more likely to have trials of labor than BNHW or NBH.

**Table 1:** Characteristics of mothers with cesarean delivery by subpopulation, US-Mexico border states, 2000–2015.

Characteristics	BH		BNHW		NBH		NBNHW	
	No.	(%)	No.	(%)	No.	(%)	No.	(%)
**Total**	458,469	(100)	125,496	(100)	1,946,498	(100)	1,551,006	(100)
**Age group (years)**								
< **20**	51,517	(11.2)	3,965	(3.2)	180,564	(9.3)	66,870	(4.3)
**20**–**34**	341,742	(74.5)	89,424	(71.3)	1,441,316	(74.1)	1,115,180	(71.9)
≥ **35**	65,210	(14.2)	32,107	(25.6)	324,618	(16.7)	368,956	(23.8)
**Education completed**^1^								
< **High school**	128,846	(34.9)	4,584	(5.3)	618,744	(40.0)	93,323	(7.7)
**High school/GED**	104,500	(28.3)	17,269	(20.0)	462,596	(29.9)	266,568	(22.0)
> **High school**	135,806	(36.8)	64,503	(74.7)	465,051	(30.1)	854,575	(70.4)
**Country of birth**^2^								
**USA**	249,100	(54.4)	112,093	(89.5)	933,228	(48.0)	1,417,958	(91.6)
**Mexico**	202,652	(44.2)	879	(0.7)	843,672	(43.4)	3,610	(0.2)
**Other foreign**	6,573	(1.4)	12,313	(9.8)	168,267	(8.7)	126,941	(8.2)
**Hispanic origin**								
**CA/SA**	5,565	(1.2)	NA	(0)	166,464	(8.6)	NA	(0)
**Cuba/Puerto Rico**	2,762	(0.6)	NA	(0)	26,206	(1.4)	NA	(0)
**Mexico**	379,554	(82.8)	NA	(0)	1,554,497	(79.9)	NA	(0)
**Other/unknown**	70,588	(15.4)	NA	(0)	199,331	(10.2)	NA	(0)
**Marital status**								
**Married**	269,032	(58.7)	100,400	(80.0)	1,047,881	(53.8)	1,188,806	(76.7)
**Unmarried**	189,437	(41.3)	25,096	(20.0)	898,617	(46.2)	362,200	(23.4)
**First prenatal care visit**^3^								
**1st trimester**	306,096	(69.8)	99,924	(86.6)	1,423,676	(76.4)	1,242,423	(84.0)
**2nd trimester**	86,658	(19.8)	11,752	(10.2)	339,354	(18.2)	183,980	(12.4)
**3rd trimester/none**	46,086	(10.5)	3,662	(3.2)	100,547	(5.4)	53,548	(3.6)
**Payment source**^4^								
**Medicaid**	107,809	(53.8)	9,334	(19.6)	530,988	(62.5)	186,067	(28.9)
**Private insurance**	45,473	(22.7)	30,652	(64.2)	244,145	(28.8)	427,024	(66.3)
**Self-pay**	18,979	(9.5)	740	(1.6)	30,963	(3.7)	10,773	(1.7)
**Other**	28,068	(14.0)	6,998	(14.7)	43,000	(5.1)	19,984	(3.1)
**State of residence**								
**Arizona**	37,431	(8.2)	26,471	(21.1)	105,279	(5.4)	141,375	(9.1)
**California**	108,666	(23.7)	79,050	(63.0)	1,158,860	(59.5)	670,276	(43.2)
**New Mexico**	14,599	(3.2)	4,825	(3.8)	38,951	(2.0)	24,921	(1.6)
**Texas**	297,773	(65.0)	15,150	(12.1)	643,408	(33.1)	714,434	(46.1)
**Gestational age**^5^								
**Pre-term**	65,981	(14.5)	18,535	(15.0)	272,909	(14.2)	239,946	(15.6)
**Early term**	152,303	(33.4)	34,702	(28.1)	576,490	(30.1)	465,198	(30.3)
**Full term**	237,439	(52.1)	70,491	(57.0)	1,066,936	(55.7)	830,725	(54.1)
**Parity**^6^								
**1st live birth**	153,060	(33.5)	55,279	(44.1)	629,146	(32.4)	648,405	(41.9)
≥ **2nd live birth**	303,974	(66.5)	69,973	(55.9)	1,315,303	(67.6)	900,354	(58.1)
**Risk status**^7^								
**Low-risk**	349,605	(79.8)	92,274	(75.1)	1,420,641	(77.7)	1,099,007	(73.7)
**Not low-risk**	88,736	(20.2)	30,525	(24.9)	408,696	(22.3)	393,238	(26.4)
**Trial of labor**^8^								
**No**	225,221	(80.2)	56,167	(83.8)	1,061,045	(83.8)	768,736	(80.0)
**Yes**	55,566	(19.8)	10,871	(16.2)	204,761	(16.2)	191,657	(20.0)

BH, border county Hispanic; BNHW, border county non-Hispanic white; NBH, nonborder county Hispanic; NBNHW, nonborder county non-Hispanic white; GED, general education diploma; CA/SA, Central America or South America.

^1^Missing values (BH = 84,883, BNHW = 36,936, NBH = 381,152, NBNHW = 325,792) occur 2003–2013 because the National Center for Health Statistics (NCHS) did not report education for unrevised birth certificates starting in 2003. Unknown not included (BH = 4,434, BNHW = 2,204, NBH = 18,955, NBNHW = 10,748).

^2^Unknown not included (BH = 144, BNHW = 211, NBH = 1,331, NBNHW = 2,497).

^3^Missing values (BH = 11,986, BNHW = 8,769, NBH = 32,766, NBNHW = 46,732) occur 2009–2013 in AZ because NCHS did not report the trimester that prenatal care began for unrevised birth certificates after 2008. Unknown not included (BH = 7,643, BNHW = 1,389, NBH = 50,155, NBNHW = 24,323).

^4^Missing values (BH = 257,003, BNHW = 77,314, NBH = 1,091,667, NBNHW = 903,538) occur before 2009 because NCHS did not report these values; payment source is only available on the revised birth certificate. Unknown not included (BH = 1,137, BNHW = 458, NBH = 5,735, NBNHW = 3,620).

^5^Unknown not included (BH = 2,746, BNHW = 1,768, NBH = 30,163, NBNHW = 15,137).

^6^Unknown not included (BH = 1,435, BNHW = 244, NBH = 2,049 NBNHW = 2,247).

^7^Unknown not included (BH = 20,128, BNHW = 2,697, NBH = 117,161, NBNHW = 58,761) due to unknown gestational age or unknown presentation. Low-risk includes births > 37 weeks of gestation, singleton birth, and vertex presentation.

^8^Missing values (BH = 151,920, BNHW = 58,085, NBH = 663,273, NBNHW = 576,526) occur 2000–2013 because trial of labor is only available on the revised birth certificate. Unknown not included (BH = 25,762, BNHW = 373, NBH = 17,419, NBNHW = 14,087). Chi-square test for BH and NBNHW trial of labor was not significant (P-value = 0.052).

**Fig 3:**
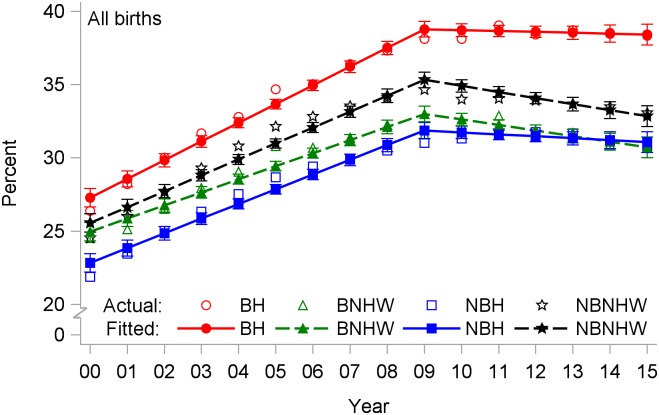
Cesarean delivery rates by year and subpopulation among all births, US-Mexico border states, 2000–2015. BH, border Hispanic; BNHW, border non-Hispanic white; NBH, nonborder Hispanic; NBNHW, nonborder non-Hispanic white.

In most years between 2000 and 2015, the overall rate of CD was lowest among NBH mothers and highest among BH mothers (Fig 3). The rate rose steadily over time until 2009 in each of the four subpopulations. The rate of CD rose fastest among BH mothers, increasing 1.27% per year from 2000 to 2009 and reaching 38.1% by 2009, 1.4 times its 2000 rate (Table 2). By comparison, peak rates in 2009 were lower for the other three groups. After 2009, the regression lines for both NHW groups declined significantly, while those for the two Hispanic groups did not.

**Table 2:** Rates^1^ and estimated annual rates of change of cesarean delivery by cesarean type and subpopulation, US border states, 2000–2015.

CD Type	Subpop.	Rate^1^ 2000	Rate^1^ 2009	Rate^1^ 2015	Pct. annual change 2000–2009 (95% CI)	Pct. annual change 2009–2015 (95% CI)
**All CD**	**BH**	26.4	38.1	38.3	1.27 (1.17, 1.38)	-0.06 (-0.23, 0.11)
	**BNHW**	24.5	31.6	30.8	0.89 (0.78, 1.00)	-0.38 (-0.55, -0.21)
	**NBH**	21.9	31.0	30.8	1.01 (0.90, 1.11)	-0.13 (-0.30, 0.04)
	**NBNHW**	24.5	34.6	33.1	1.08 (0.98, 1.19)	-0.42 (-0.58, -0.24)
**Low-risk**,^2^ **nulliparous**	**BH**	23.3	33.2	31.4	1.00 (0.88, 1.12)	-0.38 (-0.57, -0.18)
	**BNHW**	22.4	27.0	25.5	0.55 (0.42, 0.67)	-0.51 (-0.70, -0.32)
	**NBH**	18.9	26.0	24.4	0.77 (0.65, 0.89)	-0.33 (-0.52, -0.14)
	**NBNHW**	21.0	28.9	26.3	0.80 (0.68, 0.92)	-0.58 (-0.78, -0.39)
**Low-risk**,^2^ **primary**	**BH**		24.5	21.1		-0.57 (-0.74, -0.41)
	**BNHW**		19.1	16.5		-0.53 (-0.70, -0.37)
	**NBH**		16.5	15.0		-0.23 (-0.40, -0.07)
	**NBNHW**		20.2	17.6		-0.36 (-0.53, -0.20)
**Low-risk**,^2^ **repeat**	**BH**		95.3	94.6		-0.09 (-0.33, 0.16)
	**BNHW**		94.3	87.5		-1.21 (-1.45, -0.96)
	**NBH**		93.0	90.5		-0.47 (-0.72, -0.23)
	**NBNHW**		93.5	89.7		-0.65 (-0.89, -0.40)

CD, cesarean delivery; Subpop, subpopulation; Pct, percent; CI, confidence interval; BH, border county Hispanic; BNHW, border county non-Hispanic white; NBH, nonborder county Hispanic; NBNHW, nonborder county non-Hispanic white.

^1^Rates are actual rather than fitted rates per 100 live births.

^2^Low-risk births are defined as singleton, term gestation, vertex presentation births.

**Fig 4:**
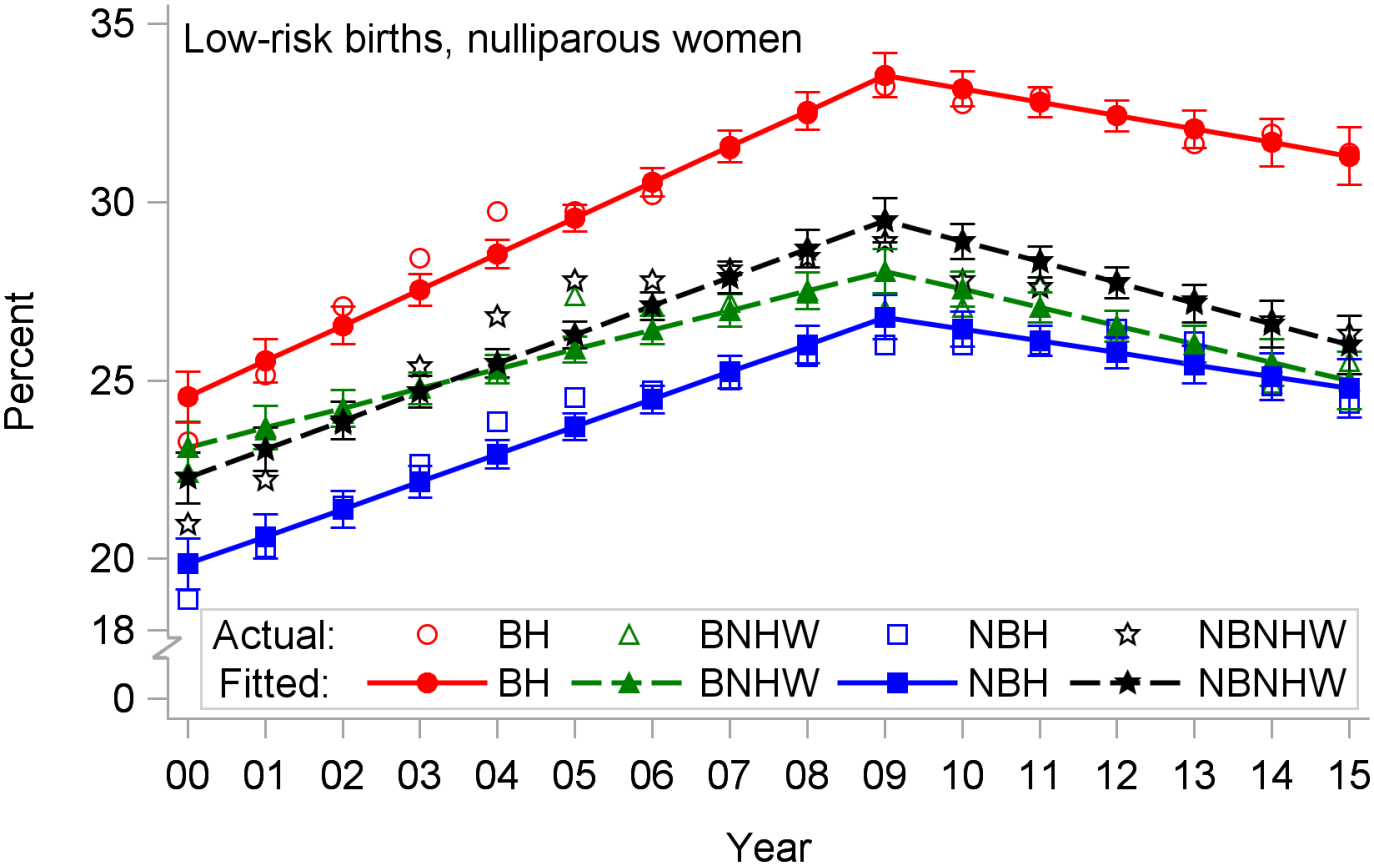
Cesarean delivery rates by year and subpopulation among low-risk births to nulliparous women, US-Mexico border states, 2000–2015. BH, border Hispanic; BNHW, border non-Hispanic white; NBH, nonborder Hispanic; NBNHW, nonborder non-Hispanic white.

A similar pattern of CD rates peaking in 2009 was found among low-risk births to nulliparous women (Fig 4). Among BH, this rate peaked at 33.2% in 2009 and declined to 31.4% in 2015. The annual rate of increase prior to 2009 was greatest among BH. Since 2009, the annual rate of decline of the low-risk nulliparous rate ranged from -0.33% in NBH to -0.58% in NBNHW (Table 2). The rates of decline were not significantly different across the four subpopulations.

**Fig 5:**
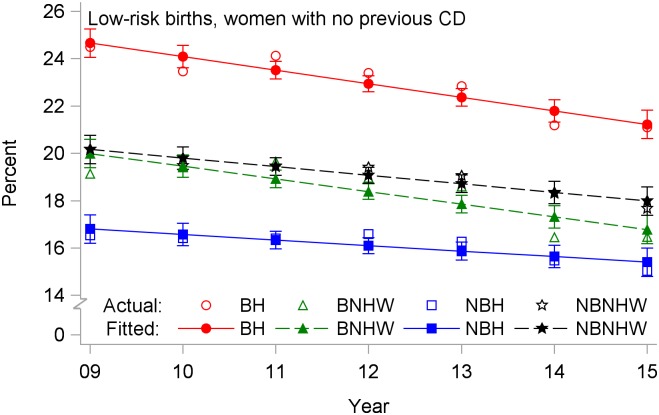
Cesarean delivery rates by year and subpopulation among low-risk births to women with no previous cesarean (primary cesareans), US-Mexico border states, 2009–2015. BH, border Hispanic; BNHW, border non-Hispanic white; NBH, nonborder Hispanic; NBNHW, nonborder non-Hispanic white.

The analysis of low-risk primary CD was limited to the years 2009 and later, where the rate fell significantly in all four subpopulations (Fig 5, Table 2). BH had the highest rate in 2009, but their rate fell -0.57% per year after 2009, significantly faster than the decline for NBH.

**Fig 6:**
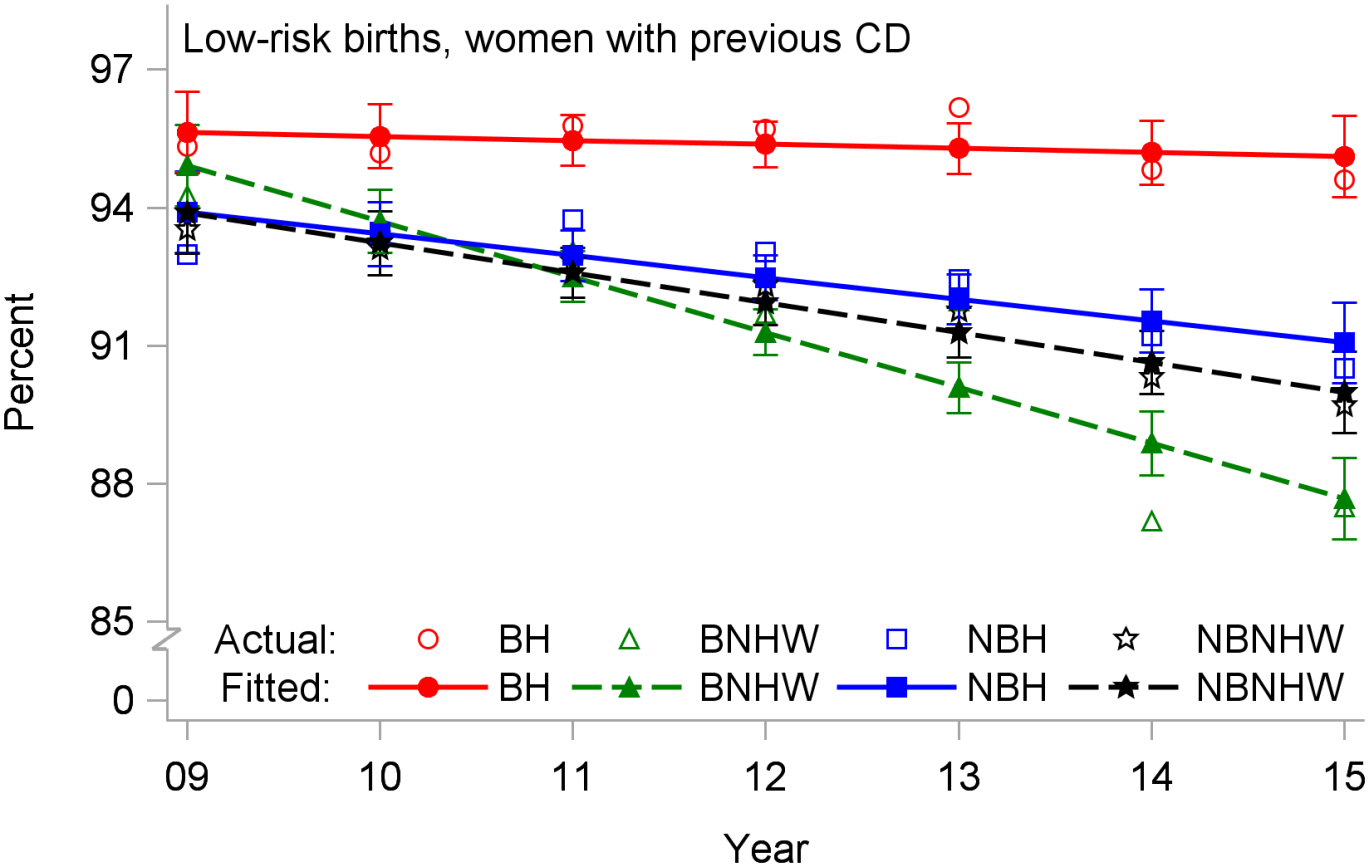
Cesarean delivery rates by year and subpopulation among low-risk births to women with a previous cesarean (repeat cesareans), US-Mexico border states, 2009–2015. BH, border Hispanic; BNHW, border non-Hispanic white; NBH, nonborder Hispanic; NBNHW, nonborder non-Hispanic white.

The rate of low-risk repeat CD (Fig 6, Table 2) did not show a significant downward trend for BH, with an annual drop of -0.09% (p = 0.64). In contrast, the average rate of repeat CD fell significantly in the other three subpopulations, with the largest decline of -1.21% per year for BNHW.

**Fig 7:**
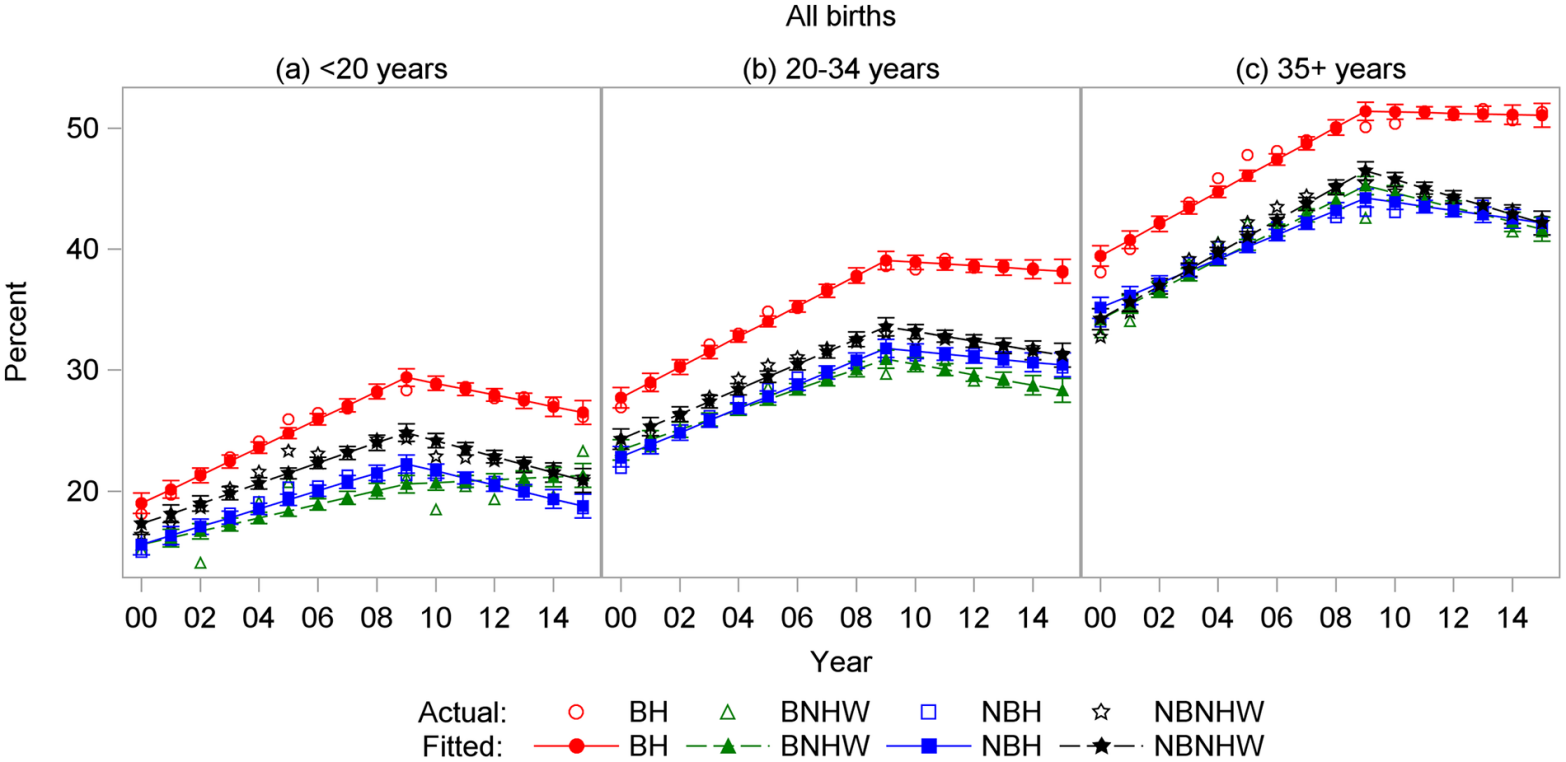
Cesarean delivery rates among all births by year and subpopulation among women ages (a) < 20 years, (b) 20–34 years, and (c) ≥ 35 years, US-Mexico border states, 2000–2015. BH, border Hispanic; BNHW, border non-Hispanic white; NBH, nonborder Hispanic; NBNHW, nonborder non-Hispanic white.

**Fig 8:**
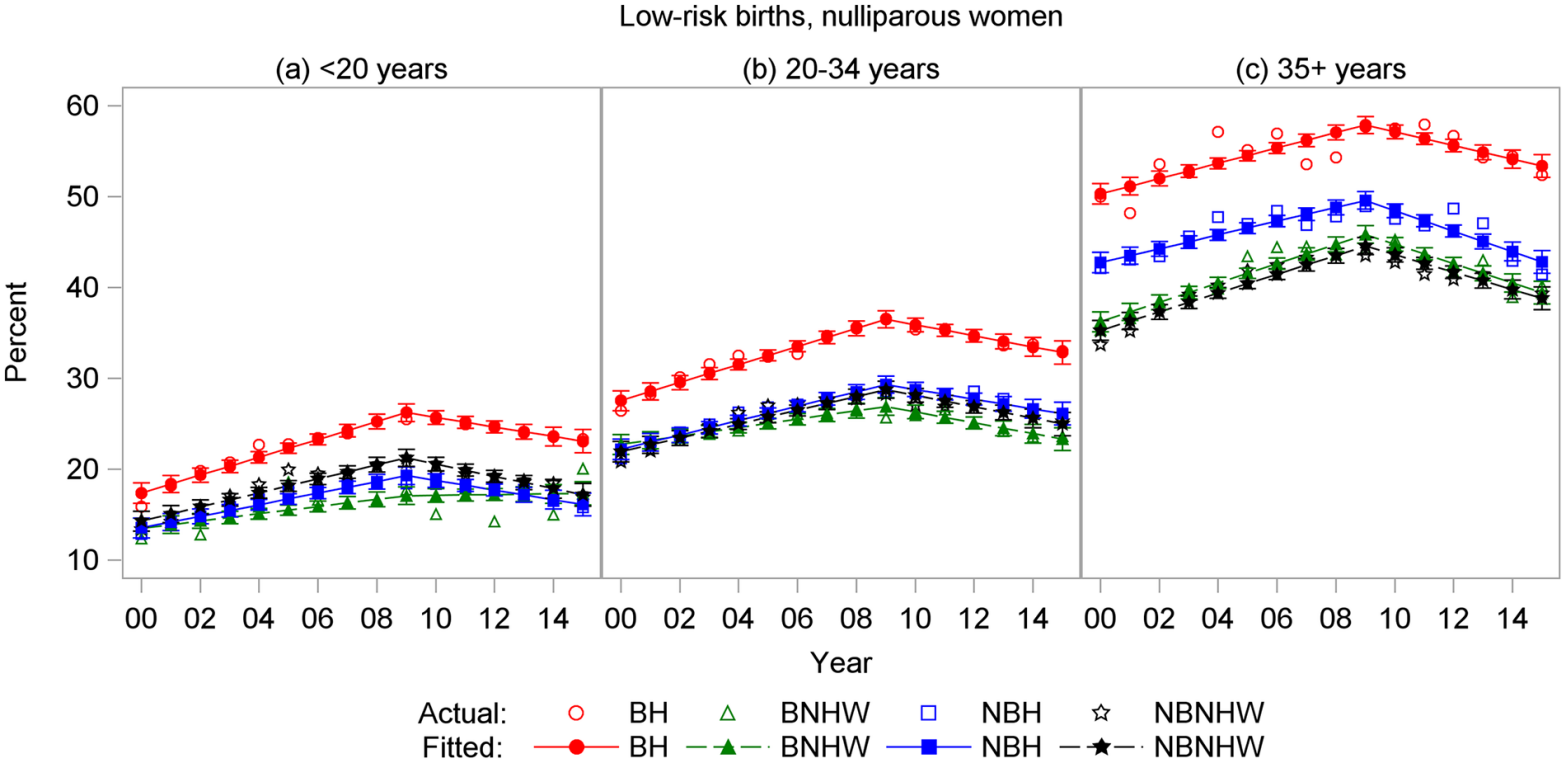
Cesarean delivery rates by year and subpopulation among low-risk births to nulliparous women ages (a) < 20 years, (b) 20–34 years, and (c) ≥ 35 years, US-Mexico border states, 2000–2015. BH, border Hispanic; BNHW, border non-Hispanic white; NBH, nonborder Hispanic; NBNHW, nonborder non-Hispanic white.

**Fig 9:**
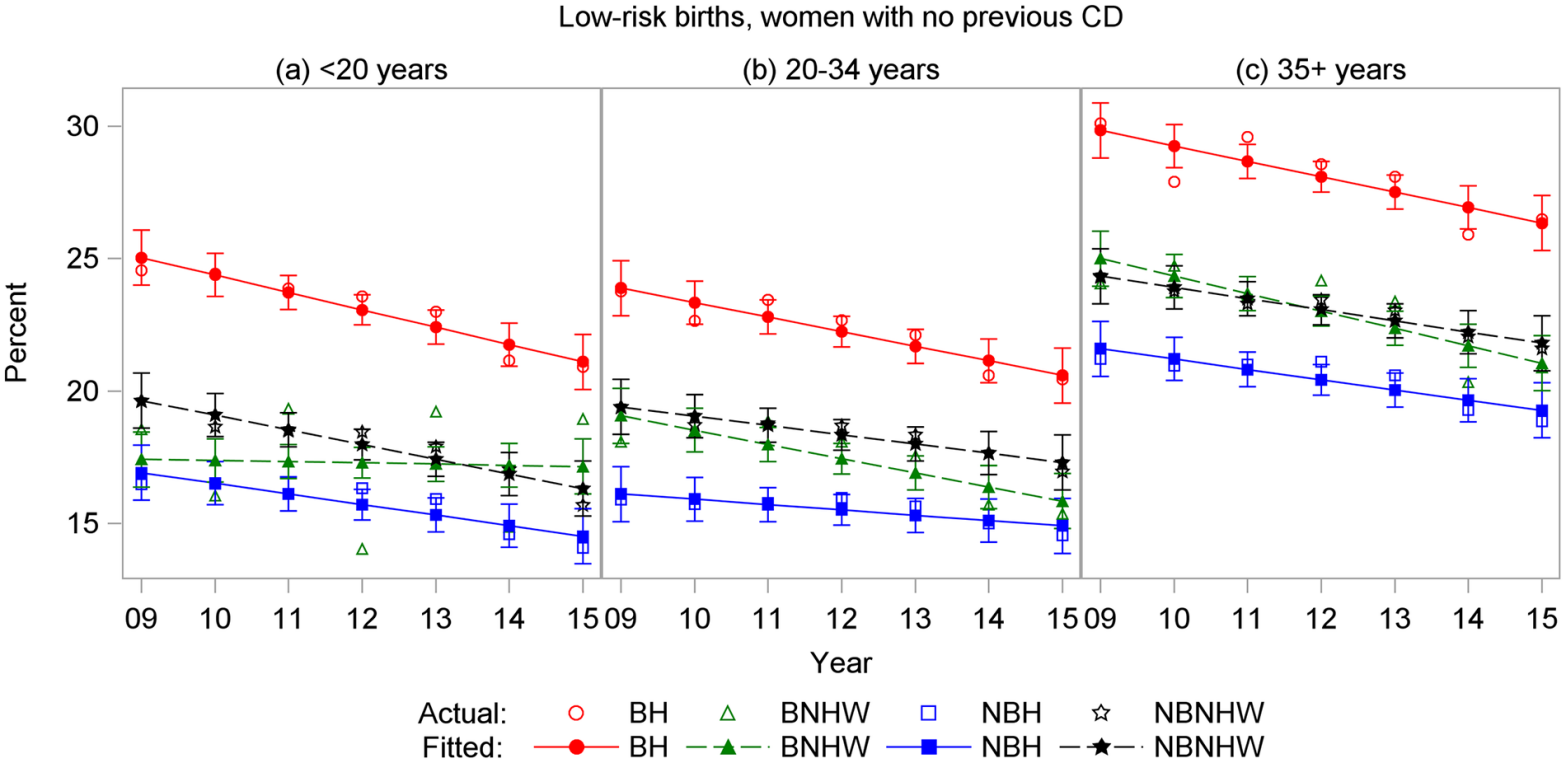
Primary cesarean delivery rates by year and subpopulation among low-risk births to women ages (a) < 20 years, (b) 20–34 years, and (c) ≥ 35 years, US-Mexico border states, 2009–2015. BH, border Hispanic; BNHW, border non-Hispanic white; NBH, nonborder Hispanic; NBNHW, nonborder non-Hispanic white.

**Fig 10:**
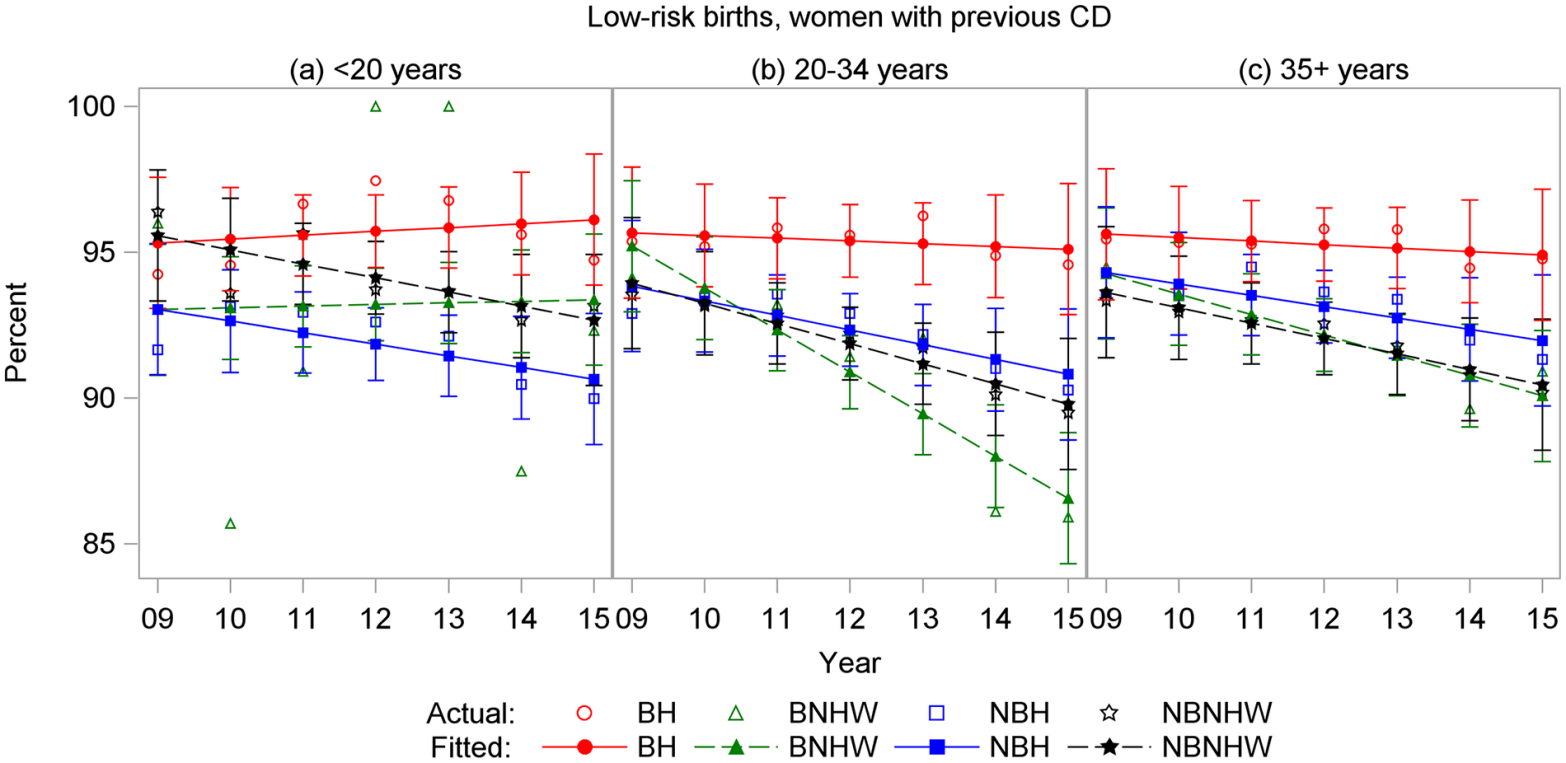
Repeat cesarean delivery rates by year and subpopulation among low-risk births to women ages (a) < 20 years, (b) 20–34 years, and (c) ≥ 35 years, US-Mexico border states, 2009–2015. BH, border Hispanic; BNHW, border non-Hispanic white; NBH, nonborder Hispanic; NBNHW, nonborder non-Hispanic white.

Age-specific rates showed similar patterns for all four CD outcomes. For total and low-risk nulliparous CD, BH rates were highest in all age groups, and rates increased with age (Figs 7 and 8). For primary CDs, rates were highest for BH and lowest for NBH in all age groups, and rates were higher in the 35+-years age group for all subpopulations (Fig 9). For repeat CDs, regression lines were flat for BH in all age groups (Fig 10).

**Fig 11:**
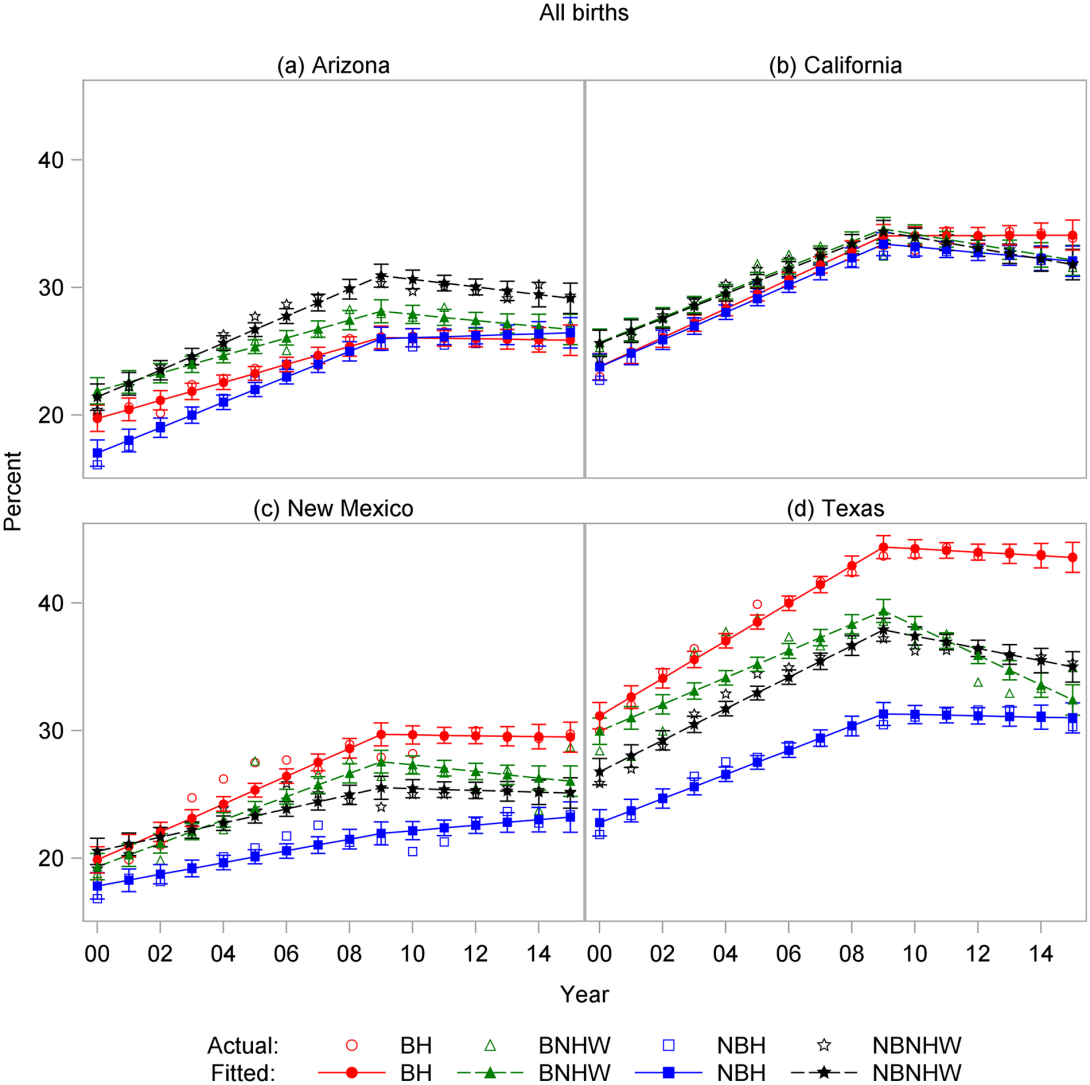
Cesarean delivery rates by year and subpopulation among all births to women in (a) Arizona, (b) California, (c) New Mexico, and (d) Texas, US-Mexico border states, 2000–2015. BH, border Hispanic; BNHW, border non-Hispanic white; NBH, nonborder Hispanic; NBNHW, nonborder non-Hispanic white.

State-specific rates for all CDs revealed marked differences (Fig 11). In Arizona, NHW rates were higher than Hispanic rates in both the border and nonborder regions, with rates for all four subpopulations <30% by 2015. In California, all rates remained between 30% and 35% after 2009, and rates were indistinguishable among the different subpopulations. In New Mexico, like Arizona, all rates were <30%, but BH rates were significantly higher than other groups after 2009. Texas had the highest state rates for BH, BNHW, and NBNHW, and rates comparable to those in California for NBH. Texas BH rates in 2015 exceeded 43%.

## Discussion

In the US-Mexico border region, NHW CD rates in border and nonborder counties peaked in 2009 and declined thereafter, in line with national data. Hispanic CD rates, however, did not conform to this trend: BH rates for all outcomes were significantly higher than rates for the other three subpopulations, and overall CD rates for BH and NBH failed to decline significantly after 2009. Comparison of low-risk primary and low-risk repeat rates shows that the failure of overall CD rates among BH to decline during 2009–2015 is at least in part due to significantly and persistently higher repeat CD rates in this group. Roughly half of the low-risk BH CDs were repeat CDs. The reason for the failure of the overall CD rate among NBH women to decline significantly is not known.

Previous work, which did not exclude non-hospital births, showed the BH CD rate in 2009 (37.9%) to be well above the US Hispanic rate that year (31.6%) [12]. The current study shows that the BH CD rate (38.3%) still exceeded the US Hispanic rate (31.7%) in 2015 and also exceeded the 2015 CD rates among US NHW (31.1%) and US blacks (35.5%). Similarly, the low-risk nulliparous rate in 2015 was 31.4% among BH versus 25.2% among US Hispanics, 24.8% among US NHW, and 29.7% among US blacks [18].

Several individual-level factors might be considered as explanations for the persistently higher BH rates. First, border Hispanics represent a population in transition from the norms of Mexico to those of the United States [12]. CD rates in Mexico are higher than BH CD rates [12]. Although border and nonborder Hispanics are equally likely to be born in Mexico, the higher BH CD rate might reflect the influence of cultural norms regarding CD among women who spend more time in Mexico than other Hispanics because they obtain prenatal care, visit family, or work across the border. Second, BH might differ demographically from Hispanics in other counties. BH mothers having CDs do have higher parity than NBH, and higher parity is a CD risk, but previous analysis showed that BH rates were higher for both nulliparous and multiparous mothers [12]. The border Hispanic maternal subpopulation is somewhat younger than the other subpopulations, but CD rates are generally lower rather than higher among younger mothers, and BH CD rates are higher in all age groups. Third, the border population is disadvantaged when compared with US Hispanics [15]. However, low income and use of Medicaid are generally associated with lower CD rates [19,20].

Hospital and other community factors might be more likely explanations for the high BH CD rates. Border hospitals are more likely to be private, for-profit than hospitals in other counties of border states [21], and the odds of CD are 50% higher in for-profit hospitals than in not-for-profit hospitals on the border [22]. Other reports also indicate higher CD rates in private, for-profit hospitals [23,24]. In addition, high rates of health care spending overall, as measured by Medicare spending, correlate with high CD rates [25]. The highest rates of Medicare spending in Texas are found in some health referral regions on the border [26], and this study shows much higher CD rates in border than in nonborder Texas counties. A previous study concluded that Texas counties were responsible for most of the BH CD rate excess [12], and Texas accounted for 65% of the BH CDs in this study.

Border community factors, however, should arguably affect Hispanics and non-Hispanics on the border equally. Indeed, in Arizona, California, and New Mexico, the BH and BNHW rates are similar, with BH rates even lower than BNHW rates in Arizona. The ethnic disparity in border counties is almost entirely driven by the rate difference in Texas for unclear reasons.

It is also unclear why low-risk repeat CD rates failed to decline among BH but dropped significantly in the other subpopulations. Perhaps language barriers and lack of access to medical records for previous CDs in Mexico make it difficult to establish the type of uterine incision used previously and other aspects of maternal history and thereby inhibit efforts to increase vaginal births after a cesarean [27]. In addition, the availability of facilities equipped to perform trials of labor after a CD has declined recently in New Mexico [28], and a lack of emergency surgical and anesthesia facilities might be a chronic problem in this medically underserved area.

This study’s strengths include the size of the study population and the fact that it is a census of all births in the border states. Consequently, this study can examine recent data on subtypes of low-risk CD, providing a first look at such CDs in the border region. The study has some limitations. First, it is a descriptive analysis of trends over time and as such does not model the contribution of individual risk factors to CD rates. Individual risk factors will be addressed in a subsequent study. Second, different birth certificate versions, using different definitions of vertex deliveries, were in use by different states during the study period. This might affect the CD categorization because low-risk births are restricted to vertex presentations. However, the National Center for Health Statistics has concluded that the national declines in low-risk CD after 2009 are not an artifact of changes in the reporting of vertex presentations [29]. Third, this analysis only includes hospital births, but non-hospital births accounted for fewer than 1% of births. Fourth, the birth certificate sometimes fails to identify risks such as breech presentation, so some high-risk CDs might have been misclassified as low-risk [30].

## Conclusions

CD rates remain much higher among border Hispanics than other US Hispanics. And no progress has been made in reducing this disparity since 2009, largely because of failure to reduce low-risk repeat CD rates. Future research into the drivers of CD disparities among border Hispanics will have to include state of residence and other contextual factors. Future research might also employ a more detailed, non-overlapping classification of CDs [31] to shed further light on the discrepancies in CD rates shown here. Reducing border CD rates will require additional interventions that address the causal factors operating in this region. Those interventions will be facilitated by additional planned analyses of patient, hospital, and community risk factors.
